# Wound Closure After Posterior Multi-level Lumbar Spine Surgery: An Anatomical Cadaver Study and Technical Note

**DOI:** 10.7759/cureus.3595

**Published:** 2018-11-14

**Authors:** Emre Yilmaz, Tamir Tawfik, Thomas M O'Lynnger, Joe Iwanaga, Ronen Blecher, Amir Abdul-Jabbar, R. Shane Tubbs, Cameron K Schmidt, Rod J Oskouian, Jens Chapman

**Affiliations:** 1 Surgery, Swedish Neuroscience Institute, Seattle, USA; 2 Neurosurgery, Swedish Neuroscience Institute, Seattle, USA; 3 Medical Education and Simulation, Seattle Science Foundation, Seattle, USA; 4 Orthopaedics, Swedish Neuroscience Institute, Seattle, USA; 5 Neurosurgery, Seattle Science Foundation, Seattle, USA; 6 Clinical Anatomy, Seattle Science Foundation, Seattle, USA

**Keywords:** wound closure, lumbar, spine surgery, fascia, technique, subcutaneous

## Abstract

Meticulous attention to wound closure in posterior lumbar spine surgery is an important principle in reducing surgical site infections. We detail standardized wound closure used for posterior lumbar spine surgery at a tertiary care referral center and illustrate this as a step-by-step cadaveric dissection. The lumbar spine of a cadaveric specimen (male, 73 years at death) was used for dissection. Standardizing wound closure in posterior lumbar spine surgery may help limit wound complications and infection. Some key points of our technique, as demonstrated on a cadaveric specimen, include separating fascial compartments, avoiding suture abscesses, and creating a tension-free wound.

## Introduction

At times neglected, wound closure is an important part of all surgeries. Postoperative wound infection has been reported in over 10% of spine surgeries, leading to patient discomfort, need for antibiotic treatment, prolonged hospitalization, and revision surgery [1-5]. However, there is no standard for wound closure after posterior lumbar spine surgery. The aim of this study was to evaluate the technical points of wound closure after posterior lumbar spine surgery.

## Technical report

**Figure 1:**
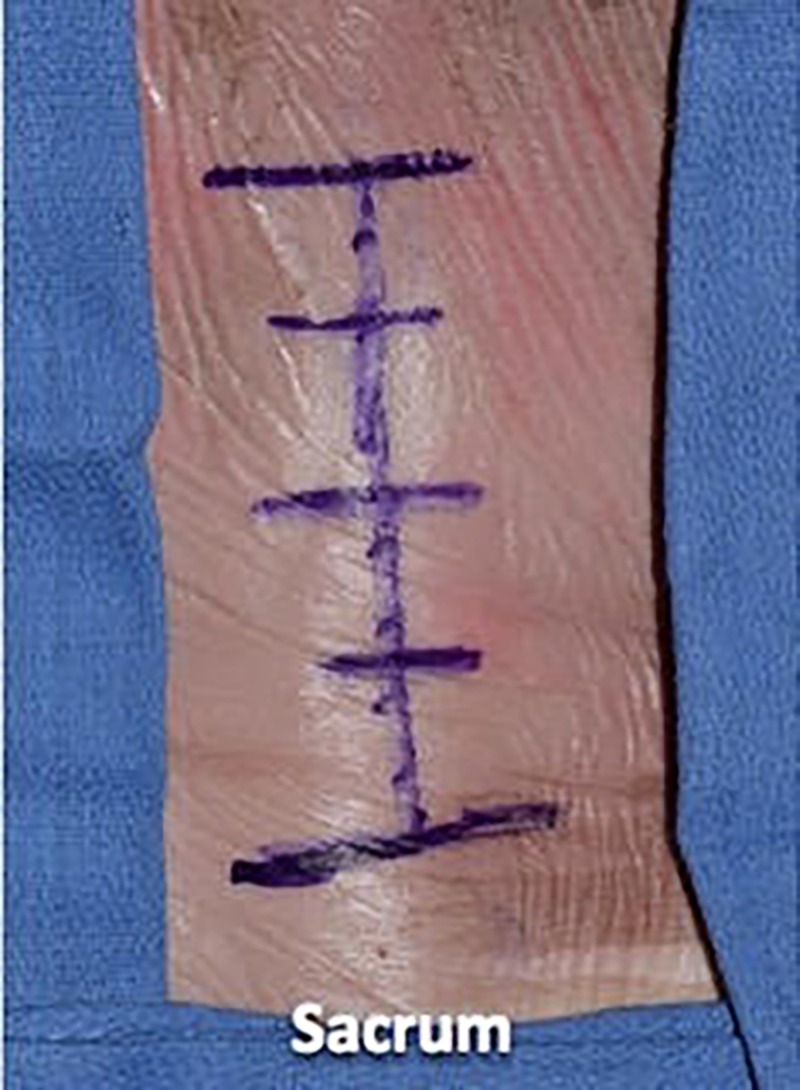
The skin of the lumbar spine is marked with a marking pen

**Figure 2:**
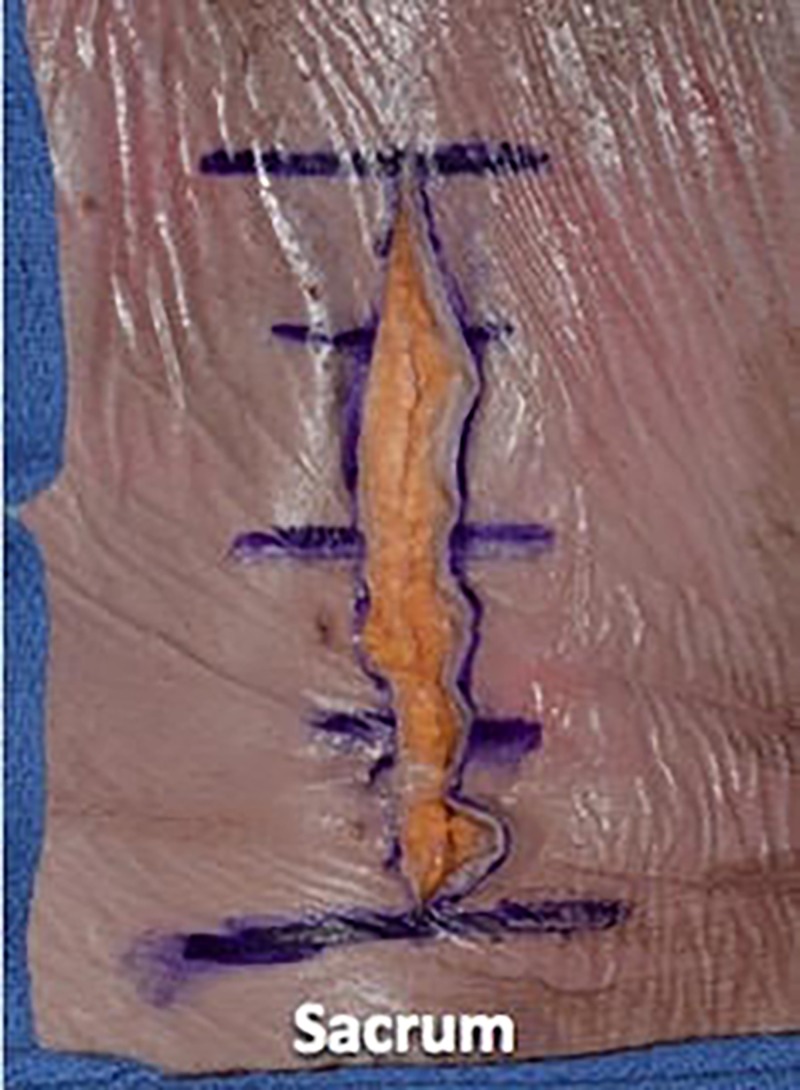
Opening the specimen sharply with a #10 scalpel blade through the epidermis and dermis

**Figure 3:**
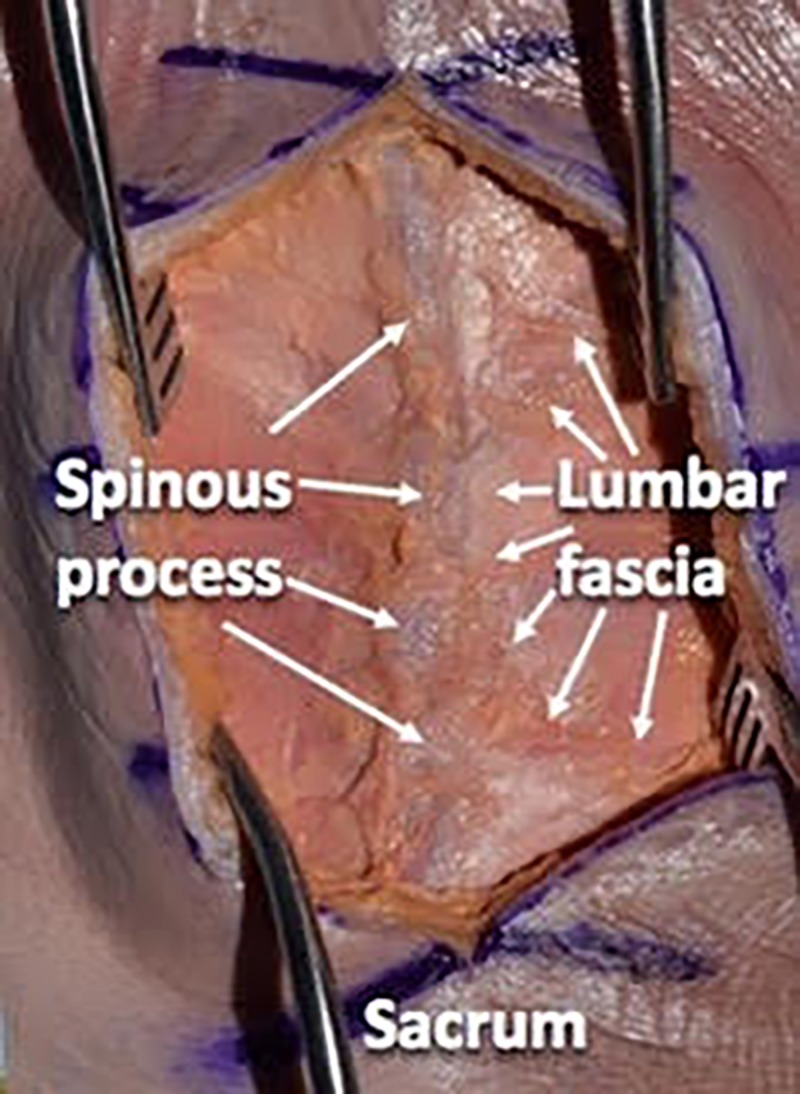
Using a Cobb elevator to separate the subdermal fat layer from the deeper fascial layer

The lumbar spine of a fresh adult male cadaveric specimen was used for dissection. In the prone position, the lumbar spine was marked with a marking pen. We then opened the specimen sharply with a #10 scalpel blade through the epidermal, dermal, and fat layers. Next, we used a Cobb elevator to separate the fat layer from the fascial layer (Figures 1-3).

**Figure 4:**
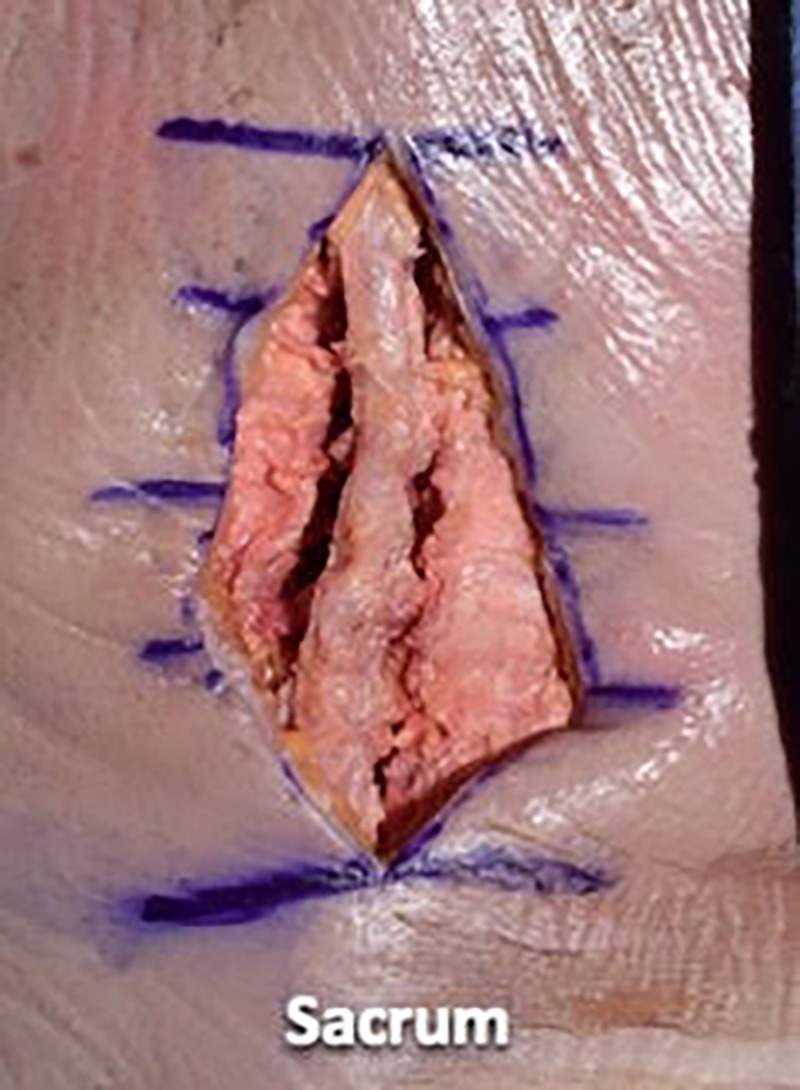
Using a #10 scalpel blade and Cobb elevator to dissect the paraspinal musculature in a subperiosteal manner from the spinous processes and laminae

**Figure 5:**
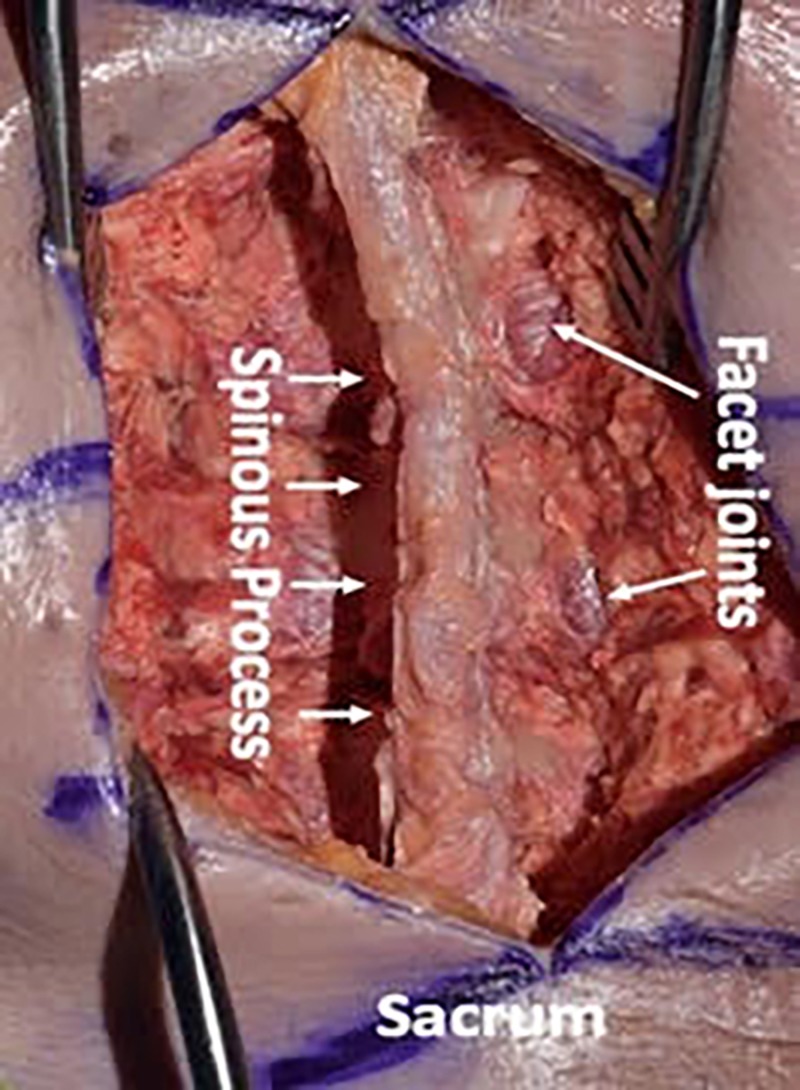
We preserved the supraspinous and interspinous ligaments during the dissection. We took the dissection up to the facet capsules without disrupting the facets

**Figure 6:**
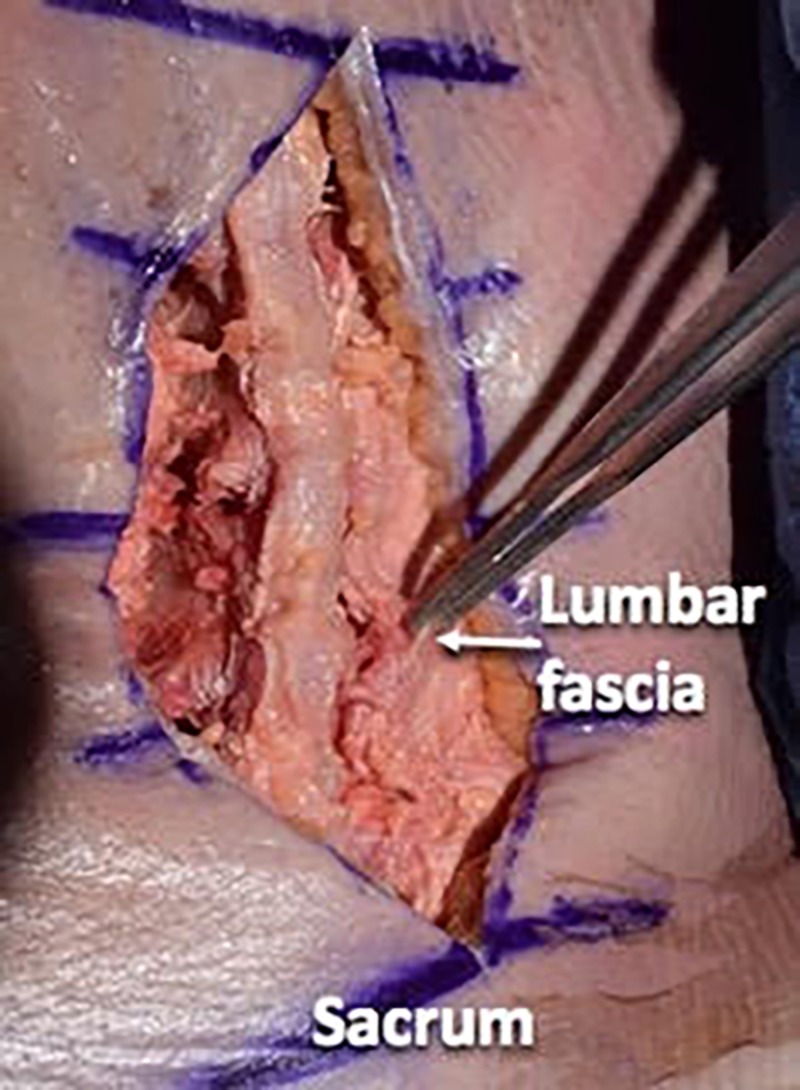
Illustrating the lumbar fascial layer

We then used a #10 scalpel blade and a Cobb elevator to dissect the paraspinal musculature in a subperiosteal manner from the spinous processes and laminae. We preserved the supraspinous and interspinous ligaments during the dissection. We took the dissection up to the facet capsules without disrupting the facet joints (Figures 4-6).

**Figure 7:**
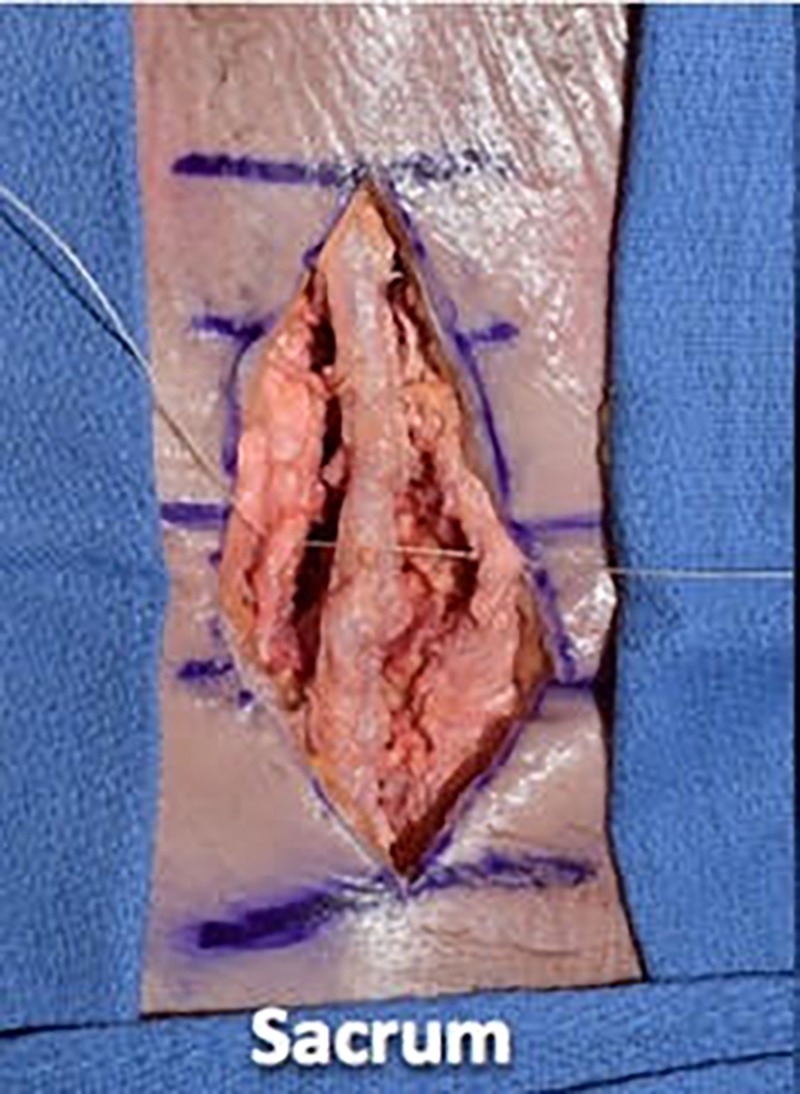
We then proceeded with wound closure, first using 0 vicryl suture to tightly reapproximate the fascial layer using a simple interrupted technique

**Figure 8:**
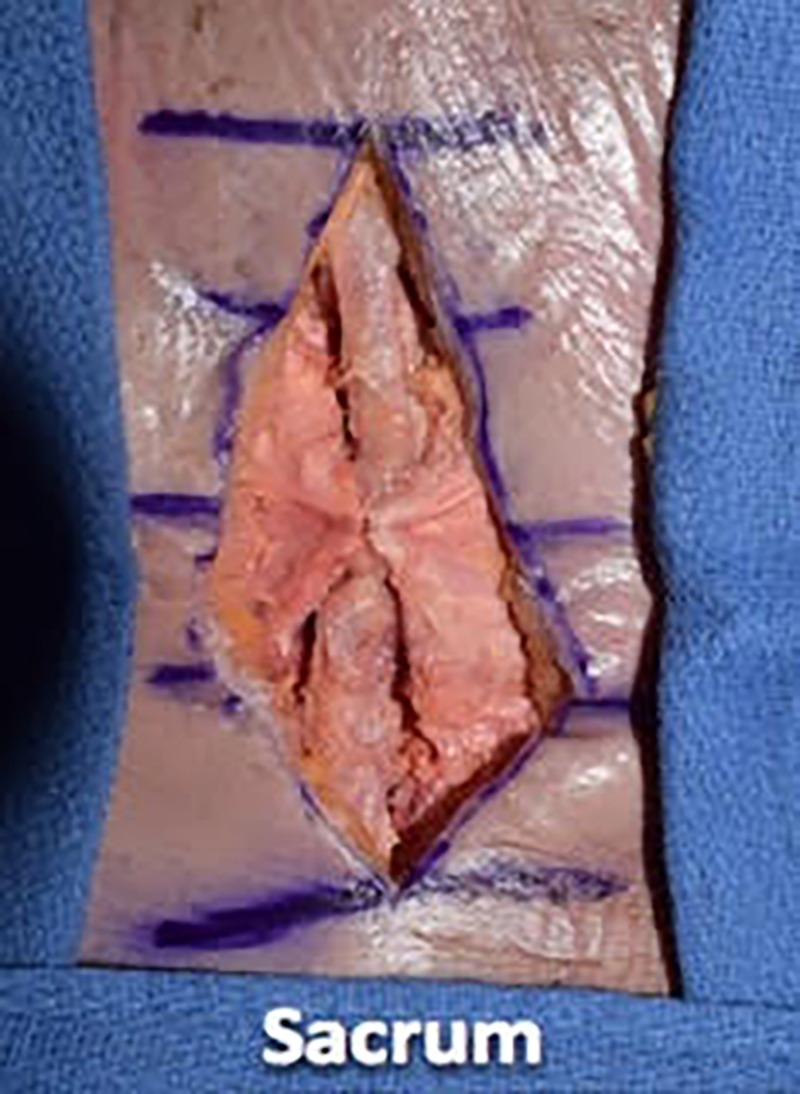
Illustrating the single interrupted fascial closure technique

**Figure 9:**
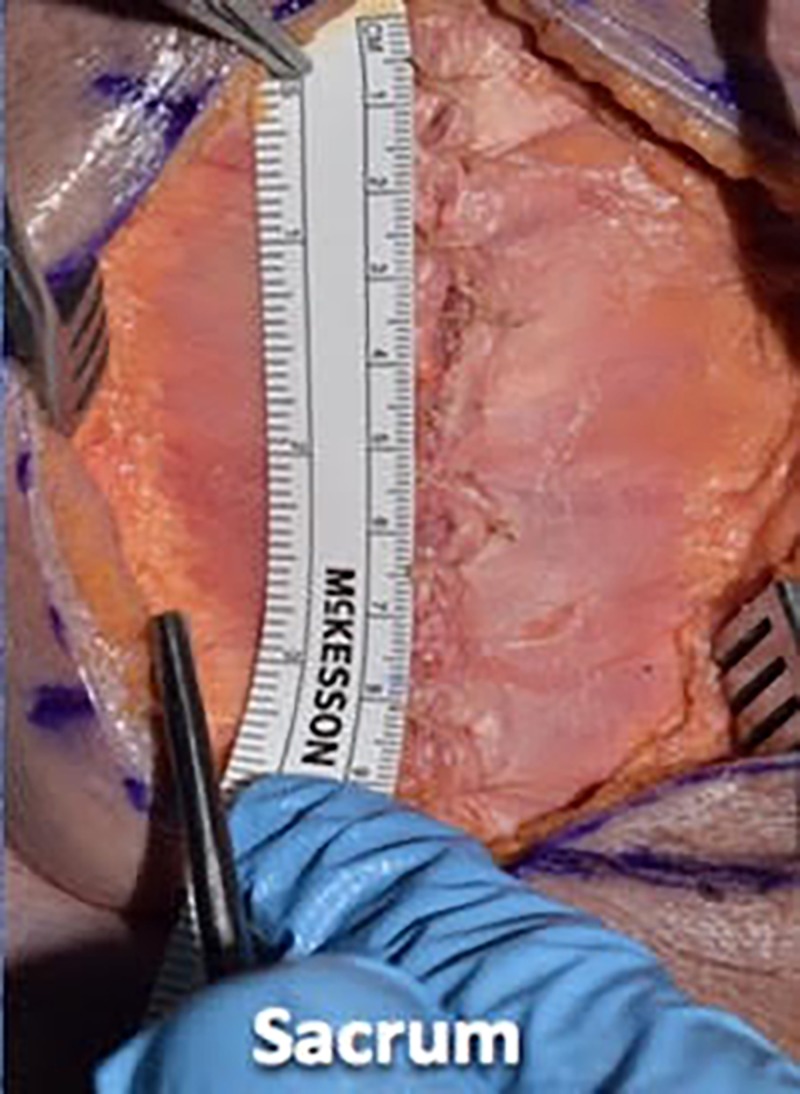
We placed sutures in approximately 1 cm increments and tied four square knots to lock each suture down

We then proceeded with wound closure by first using a 0 vicryl suture to tightly reapproximate the fascial layer using a simple interrupted technique. We placed the suture in approximately 1-cm increments and tied four square knots to lock each suture down (Figures 7-9).

**Figure 10:**
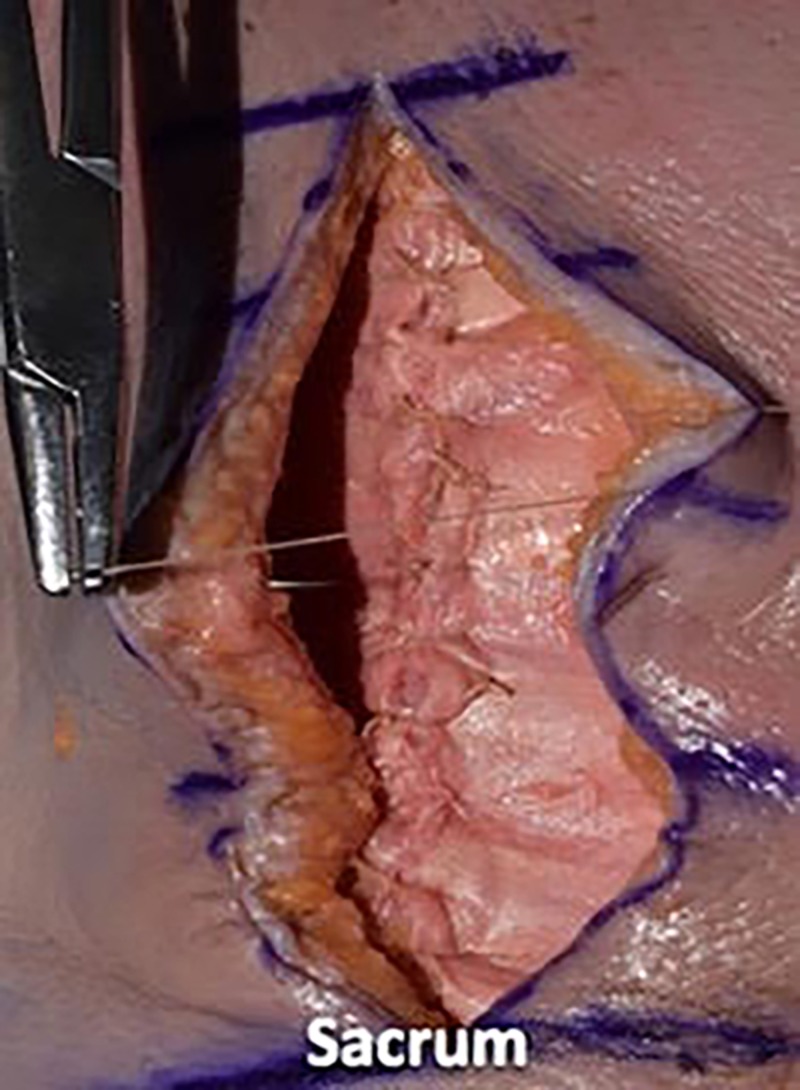
We then utilized 2-0 vicryl suture in an inverted manner to close the dermal layer with buried knots, avoiding entry into the epidermal layer

**Figure 11:**
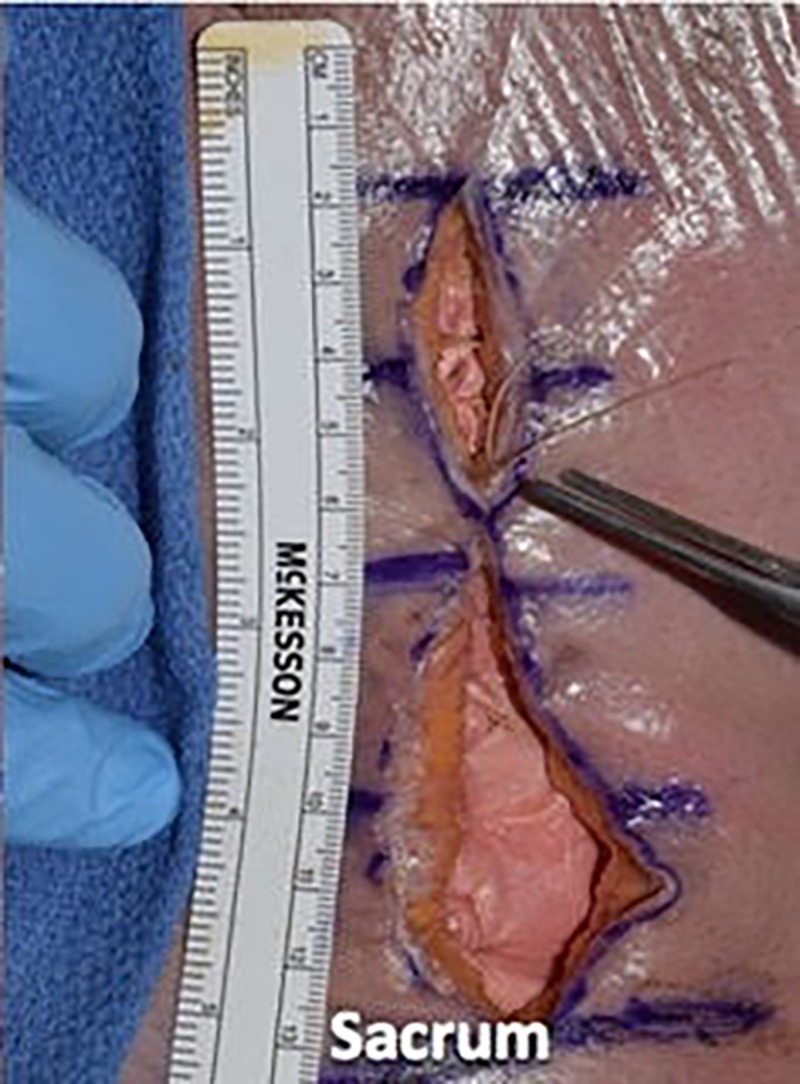
We spaced each of these sutures in 1-cm increments and tied four square knots

**Figure 12:**
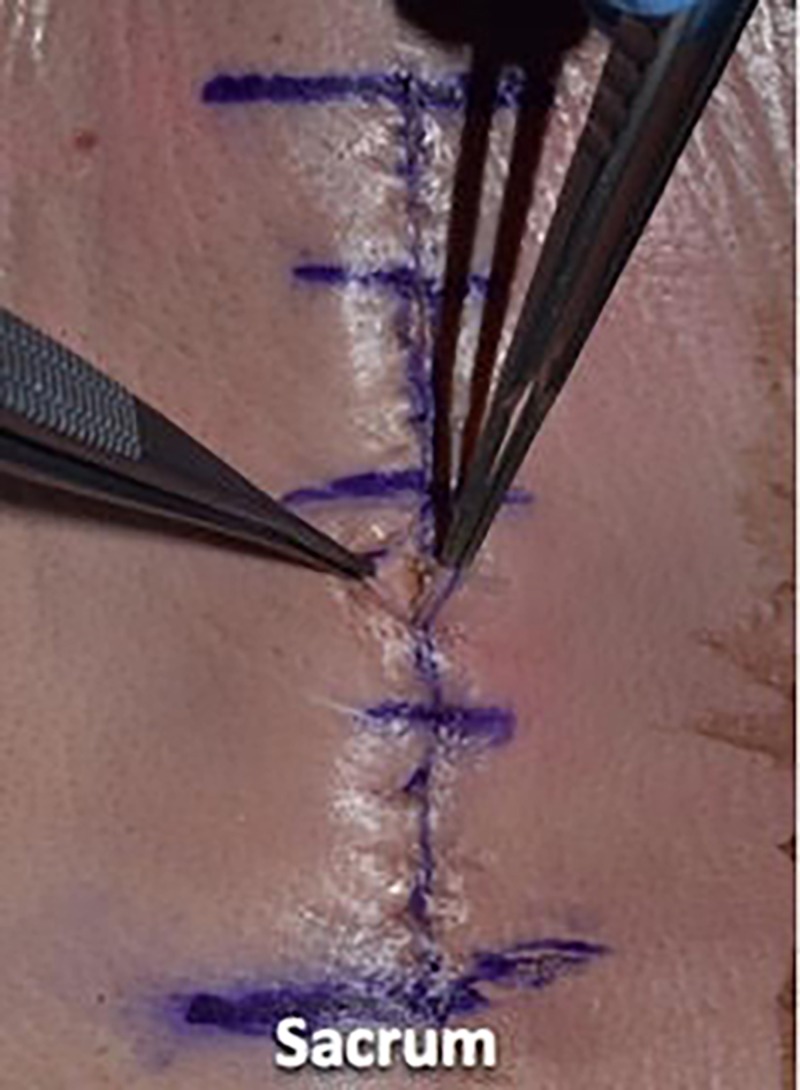
Illustrating the inverted dermal closure

We then utilized a 2-0 vicryl suture in an inverted manner to close the dermal layer with buried knots, avoiding entry into the epidermal layer. We spaced each of these sutures in 1-cm increments and tied four square knots (Figures 10-12).

**Figure 13:**
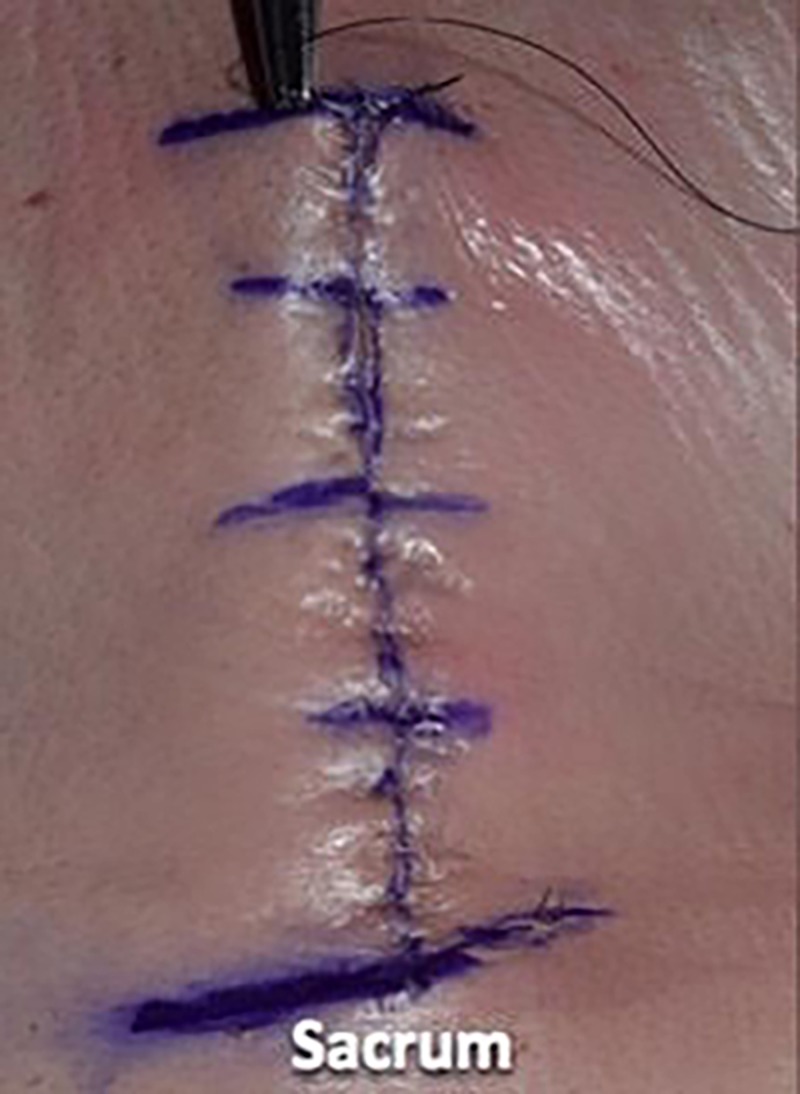
We then utilized 3-0 nylon suture in a simple interrupted fashion to reapproximate the skin

**Figure 14:**
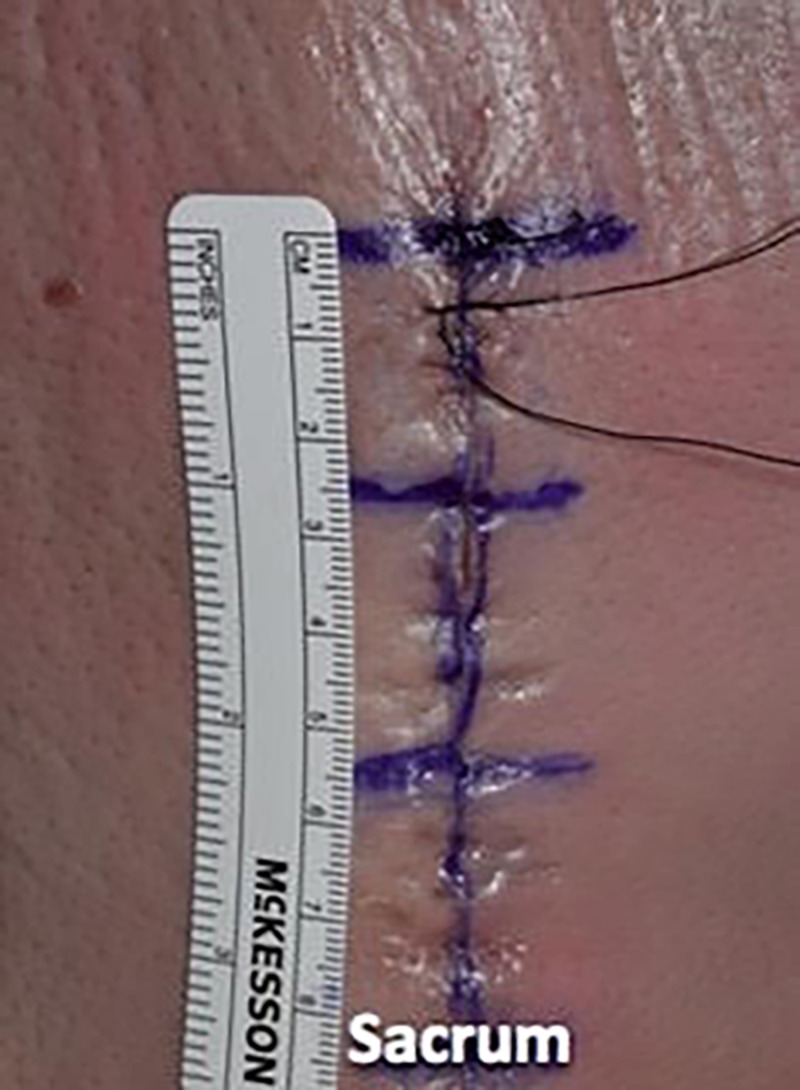
The sutures were placed in 1-cm increments

**Figure 15:**
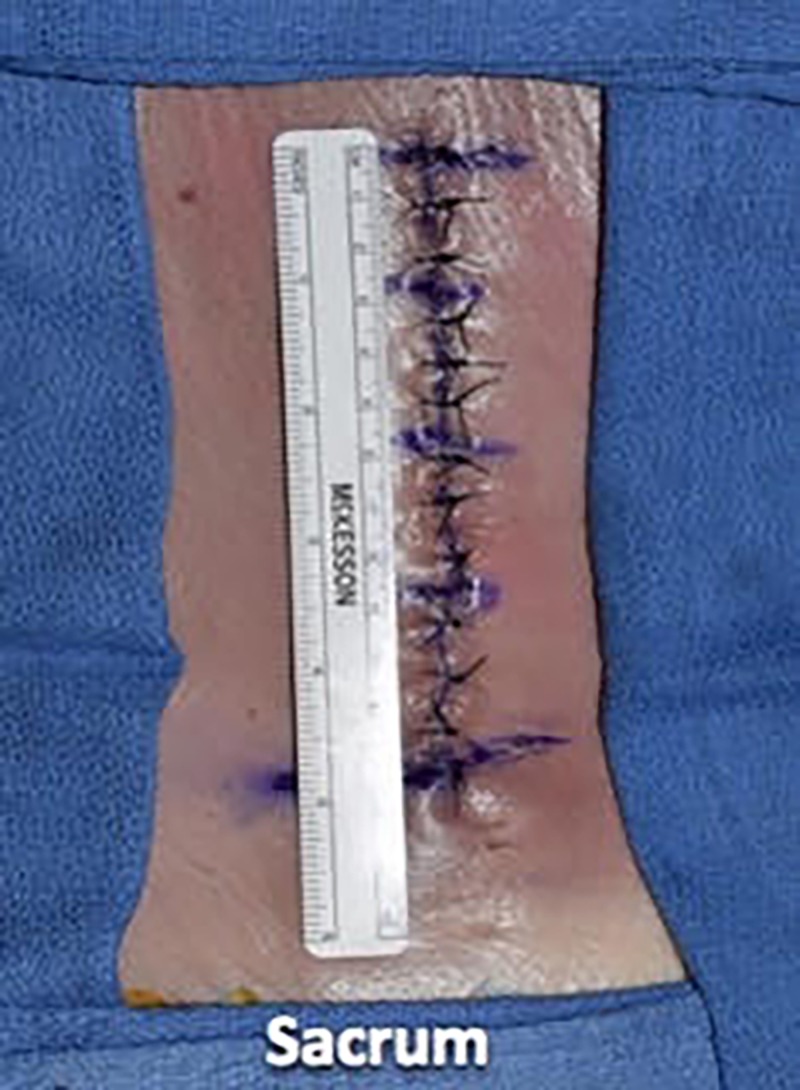
Illustrating the epidermal wound closure

We then utilized a 3-0 nylon suture in a simple interrupted fashion to reapproximate the skin, placing the suture in 1-cm increments and using eight knots for this monofilament suture (Figures 13-15).

## Discussion

Postoperative wound complications in posterior lumbar spine surgery cause significant morbidity and increase costs [1-5]. Standardizing wound closure may help to prevent patient harm and save money. In this article, we set forth the standardized wound closure used at a tertiary care referral center, as demonstrated on a cadaveric specimen. Some important points to consider in wound closure are separating fascial compartments, preventing stitch abscesses, and creating a tension-free wound. By identifying the thoracolumbar fascia and carefully dissecting it out prior to paraspinal muscle dissection, it is easier to identify this layer at closure. This allows for a tighter reapproximation of the fascial plane without including adipose tissue, which could weaken the suture. By separating the fascial compartments tightly, there may be less risk of seeding a superficial infection into the deeper compartment, which may contain hardware [6]. Stitch abscesses may occur when suture material is too close to the surface, resulting in an inflammatory reaction and expulsion of the suture material. This local reaction may contribute to infection. By inverting the dermal suture, avoiding excessive knots, and keeping the suture bites below the level of the epidermis, the risk of stitch abscess may be reduced [7]. Creating a tension-free wound may aid in wound healing. Tension on the superficial wound can contribute to vascular compromise and the resultant skin necrosis [6]. Spacing stitches too close together may also compromise blood flow between sutures and cause tissue strangulation. Using simple interrupted sutures results in greater tensile strength as compared to a running stitch [8].

## Conclusions

Standardizing posterior lumbar spine surgery wound closure may be an important way to help reduce wound complications that can have a significant impact on patient outcomes and healthcare expenditure. Meticulous attention to closure and limiting variation in technique has the potential to limit wound dehiscence and infection, reducing the need for antibiotics and a return to the operating room.
